# Deep recurrent infection of the hip after tumoral resection in an 18–years old male–a case report

**Published:** 2008-11-15

**Authors:** M Niculescu, M Negrusoiu

**Affiliations:** Orthopedic Surgery 1, Colentina Clinical Hospital, BucharestRomania

## Abstract

An 18 years old male was referred to us 4 years after major tumoral surgery. In 2002 he was diagnosed with Ewing sarcoma of the proximal two–thirds of his left femur. Wide resection of the tumor was performed, with a makeshift implant one–stage reconstruction, followed by a combination of chemo and radiotherapy for another 6 months.

Eight months after surgery a deep infection of the hip developed, and despite antibiotic treatment and two consecutive debridments and lavage the results were negative. When we first saw the patient in 2006, he still had an active infection in his left hip and a septic general appearance. A two–stage revision was performed and a modular tumoral reconstruction was implanted. At two years follow up the patient presents no signs of recurrence neither of the infection nor of the primary tumor.

Replacement of bone segments after resection of a tumor–containing bone has a long history, and currently metallic implants and allografts have reasonable rates of success in terms of patient survival and restoration of useful function. Large femoral resections for extensive tumors are uncommon but clearly represent a major problem, which in the past has required hip disarticulations. In recent years, resection and replacement using custom metallic implants and more recently modular devices have allowed the patients to be restored to reasonable function.

An 18 years old male was referred to us 4 years after major tumoral surgery. In 2002 he was diagnosed with Ewing sarcoma of the proximal two–thirds of his left femur. Wide resection of the tumor was performed, with a makeshift implant one–stage reconstruction.

The implant used for reconstruction was a combination of a long (275mm) Restoration DLS (Stryker Inc.) hip revision stem and a supracondylar nail. The DLS stem has two distal holes for femoral fixation. Two similar holes were drilled in the proximal end of the supracondylar nail and the two of them were bolted together. The distal part of the nail was secured in the remaining distal femur with intercondylar screws. A cemented acetabular cup was used (Fig. 1).

**Fig. 1:**
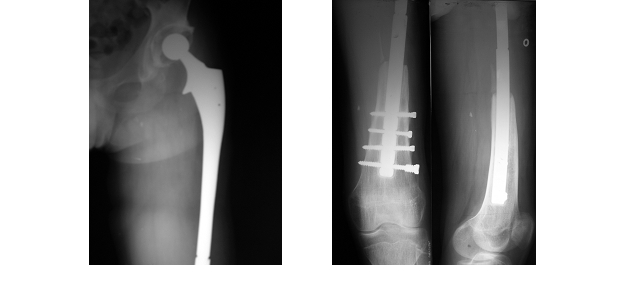
Initial reconstruction after tumoral resection

The reconstruction was successful, with preservation of the limb length and function, with partial weight–bearing three days after surgery and full weight bearing allowed at 4 weeks.In the mean time, a 6 months oncologic treatment involving chemo and radiotherapy was carried on.

Initial results were encouraging, but at eight months after surgery signs of deep infection occurred. Despite of early antibiotherapy the infection progressed to an open active fistula in the proximal part of the hip. A large debridement and lavage was done leaving the implant in place but with scarce results. Due to the recurrence of the infection a second debridement was done 18 months later, but still with no results. In both cases Coagulase–negative Staphylococcus was isolated from the site.

After the second failed attempt to clear infection, a hip disarticulation was proposed to the patient and the family. They refused and were referred to our department for a second opinion.At admittance, the patient presented with an open fistula of the proximal part of the hip from which small amounts of latescent liquid drained. His general state was one of a chronic infection. Biologically he had high WBC count, elevated ESR (75/h) and 3–times above normal CRP. Procalcitonine test was positive.

The A–P and lateral X–rays of the hip showed the femoral implant in place, with no signs of motion in the distal femur, but with radiolucent lines all around the acetabular cup (Fig. 2).

**Fig. 2:**
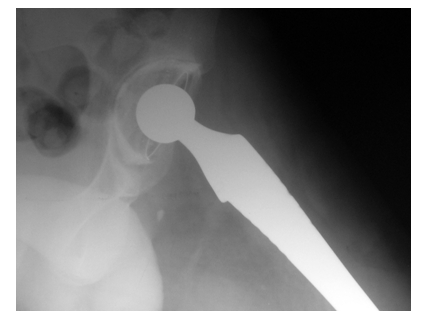
Radiolucent lines around the cup showing septic loosening

Coagulase-negative Staphylococcus was isolated from the site.

After complete evaluation of the patient a two–stage revision–reconstruction of the hip was decided. The reasons for choosing these procedures were the age of the patient, the strong denial of a hip disarticulation and the qvasi–benign nature of the germ involved. The arguments against this procedure were the duration of the infection (3 years), previous failed surgeries and the lack of an appropriate hip spacer, which was the most acute problem we had.

An external fixator was quickly excluded because of the large gap it had to cover and the presence of infection which could be maintained or worsened by subsequent pin site secondary sepsis, so we decided to use the old implant.

In surgery an extended approach from the lateral aspect of the hip to the lateral side of the knee was performed. The acetabular cup and cement was removed along with the femoral implant. Large debridement was done followed by abundant (20L) saline lavage. The femoral implant looked stable and after thorough cleansing it was autoclaved for 45 min at 132^Ŷ^C. After lavage Betadine swaps were left in place for 25 minutes followed by another abundant lavage. A new acetabular cup was inserted using antibiotic medicated cement and the femoral component was reinserted. A passive drainage was kept in place for 48 hours.

After surgery a 6 weeks antibiotic regimen followed–Teicoplanin 400mg q.d. combined with Rifampicin 300mg b.i.d.

The postoperative evolution was good, with primary healing of the wound and the normalization of the laboratory tests at 8 weeks.

The patient was discharged free of infection awaiting the three months free interval to the final reconstruction. 

Unfortunately, after 2 months, the femoral implant broke were its components were bolted together, so the patient was placed in a hip cast with no weight bearing until final surgery.

After one month there were no signs of septic recurrence and the final reconstruction with a HMRS (Stryker Inc.) femoral system was performed (Fig. 3).

**Fig. 3:**
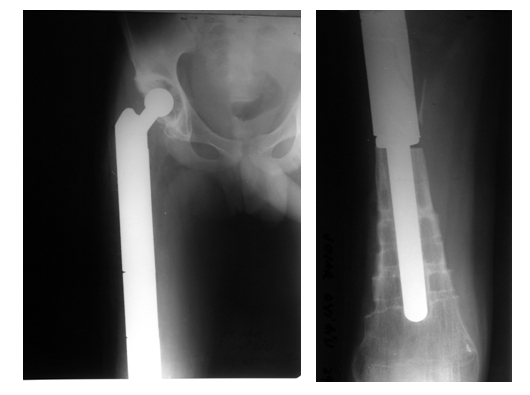
Final reconstruction with the HMRS system

At one year follow up the patient has no signs of recurrent sepsis and the hip function is 91 according to Harris Hip Score. No primary or secondary malignancies were noted

**Discutions**

Prosthetic replacement following excision of a bone tumor can be complicated by infection because patients who undergo surgery for a neoplasic condition often are subjected to extensive soft–tissue dissection and long operating times and are immunosuppressed.

The epidemiology, risk factors, and efficacy of therapy for infections complicating limb–sparing surgery (LSS) are not understood completely. The treatment of infection in these patients is arduous and lengthy, with a substantial risk of amputation.

Gaur and al. conducted in 2005 a study on one hundred three patients which underwent 104 LSS procedures. Infections at the LSS site occurred in 26% of patients, and 21% of patients developed orthopedic device infections (ODIs), which greatly surpass the infection rates for conventional joint replacement.[3]

Coagulase–negative Staphylococcus and Staphylococcus aureus are the most frequently involved in this cases.(94%) 

Studies in the literature suggest that two–stage revision can successfully treat the infection in 72% of cases, but amputation to treat the infection go as high as 36%.[4,5]

Antibiotics directed at all significant pathogens are required, ideally those with good activity against adherent bacteria and those producing a biofilm, e.g. rifampicin or one of the fluoroquinolones. ODI requires antibiotic treatment for >6 weeks to several months in addition to surgery. [1]

The effect of rifampicin in combination with various antibiotics has been very encouraging in clinical trials despite in vitro synergy and time–kill studies, which might appear to contradict this.[9] It is particularly useful in eradicating bacteria adherent to prosthetic material in joint infection or chronic osteomyelitis. 

Rifampicin has excellent anti–staphylococcal activity and bioavailability, can penetrate white blood cells to kill phagocytosed bacteria and can eradicate adherent organisms in the stationary phase making it the (almost) ideal antibiotic for bone infection. It has been shown to be particularly successful as an adjunct in PJI or osteomyelitis with metal pins in situ.[6, 8]

**Conclusions**

Patients treated with an orthopaedic procedure for an oncological condition have high infection rates. The treatment of infection in these patients is arduous and lengthy, with a substantial risk of amputation.

Current treatment for bone malignancies is complicated by an unexpectedly high incidence of infection. ODI is the most common reason for amputation and poor functional outcomes. The identification of risk factors for ODI may allow modifications of therapy that reduce the incidence and severity of infection, but prevention of all ODIs will require novel strategies.
